# Factors Supporting Implementation among CDSMP Organizations

**DOI:** 10.3389/fpubh.2014.00237

**Published:** 2015-04-27

**Authors:** Deborah Paone

**Affiliations:** ^1^Paone & Associates, LLC, Minneapolis, MN, USA

**Keywords:** implementation, evidence-based health promotion, organizational capacity, implementation factors, sustainability, chronic disease self-management program

## Abstract

Reaching individuals who can benefit from evidence-based health promotion and disability prevention programs is a goal of federal, state, and local agencies as well as researchers, providers, community agencies, and other stakeholders. Implementation effectiveness at the organizational level must be achieved in order to reach these individuals and sustain the program. This mixed methods study examined eight organizations within two states that successfully implemented the Chronic Disease Self-Management Program (CDSMP) and sustained it from 4 to 10 years. There were two types of organizations: aging services and health care. Internal and external implementation factors and influences were explored. Additional examination of state activities (as a key external agent supporting CDSMP implementation) was conducted. The examination found agreement among the eight organizations regarding why they had adopted the CDSMP – citing the alignment between the program and their organizations’ mission and purpose to improve health status and promote better self-care, and the demonstrated value (benefits) of the program. Organizations were also alike in that they described the importance of an internal champion and supportive senior leader. Organizations differed in how they experienced and valued peer support and collaborative networks. Organizations also differed in how they filled their CDSMP workshops. Internal drivers and capability were more often discussed as facilitating successful implementation than external factors. However, state activities and external support enabled successful adoption – particularly funding and training. The primary challenges identified by this set of organizations included difficulty in recruiting participants (filling workshops) and irregular or insufficient funding sources. These challenges were identified as significant and represented barriers to sustaining the program.

## Introduction

Reaching individuals who can benefit from evidence-based health promotion (EBHP) and disability prevention programs is an important goal for public health. Stakeholders for successful EBHP program dissemination and implementation include: the individual/consumer, program manager or champion within an organization, the organizational executive, a purveyor or external agent such as the state department of health or of aging, funding organizations, national program centers maintaining fidelity-monitoring, federal agencies, and policymakers. Decisions made at each level can change the landscape for effective implementation.

Evidenced-based programs can be viewed as complex innovations – those requiring multiple inputs within an organization or system. The path from adoption to sustainability of evidence-based programs is often characterized by a series of fits and starts, with internal and external forces affecting progress. For example, Fixsen, Blasé, and colleagues place special emphasis on *human capability and systems* [emphasis added] that support the practitioner/worker implementing the program (1). Because implementation is so dependent on human behavior, successful and sustained implementation will require ongoing training, coaching, feedback, data, and other systems working in tandem to regularly maintain the desired behavior [(1), p. 4]. Durlak and DuPre focus on environment/context and implementation structure and factors that influence the implementation process including: community participation/collaboration, provider characteristics, innovation characteristics, organizational capacity, and technical assistance/training (2). Greenhalgh and colleagues describe a good “innovation to system fit” as a key factor where the existing values, norms, strategies, goals, skill mix, supporting technologies, and ways of working are aligned (3).

Other internal factors influencing implementation success at the organizational level include: organizational leadership, organizational climate, staff capability, staff buy-in, and acceptability to the consumer, patient, or client (4–8). External factors found to be important to include: technical assistance and availability of adequate resources (9, 10). Community-based organizations, in particular, may have additional challenges or constraints requiring adaptation to the type or level of technical assistance, or to the protocol itself (10, 11). For example, one study of community-based organizations found that barriers to EBHP program adoption included: resource constraints, program adaptation challenges, and conflicts with organizational culture (12).

### Chronic disease self-management program

The Chronic Disease Self-Management Program (CDSMP) is an evidence-based program for adults with chronic disease to encourage these individuals to better manage and maintain their health status. The development of the CDSMP evolved from knowledge and practice experience gained from the Arthritis Self-Management Program [(13), p. 680]. The CDSMP is designed to build on the strengths and capability of individuals – including belief in their own abilities, knowledge of what to do regarding their condition, and behavior skills to address situations that arise (13). The program was tested in a randomized controlled trial of 952 subjects receiving the CDSMP from community-based program sites in the 1990s followed by another study of 831 subjects followed over several years through a longitudinal trial. In both trials, CDSMP proved to have significant positive effects on participants’ self-efficacy, levels of exercise, self-reported health, and other health status measures (14). The participant group also had fewer hospital days (14, 15).

One estimate puts a dollar value of potential medical care cost savings at over $4.2 billion – savings that could be realized from better health management if just 10% of persons with chronic conditions participated in the CDSMP (16, 17). In addition to the medical cost savings, there are quality of life benefits for individuals who are more actively engaged in their health management. A study of the health-related outcomes of a sample of 687 CDSMP participants found that significant improvements were observed for health outcomes such as depression, self-assessed health, and unhealthy physical days (18).

The CDSMP follows a 6-week, 2.5 h/week group format and is guided by a tightly scripted protocol that is delivered by certified instructors. Each week, the workshop focuses on a specific self-care management and educational topic. Instructors (two instructors are required for every workshop) follow guidelines and participants set a goal each week to pursue. Participants report on progress they have made, week by week, to the other participants in the group. The format includes facilitated interaction and group sharing. Participants often encourage each other and offer insights into the way they have managed their own conditions.

Dissemination of the CDSMP was fostered through a collaborative initiative (called “Communities Putting Prevention to Work: CDSMP”) funded under the American Reinvestment and Recovery Act (ARRA). Two-year grants (2010–2012) totaling $27 million were awarded to 45 states. A program evaluation of this national dissemination initiative for CDSMP was conducted in 2012–2013 (19). This process evaluation focused primarily on the states’ (purveyors’) activities. The state units on aging and the state or local public health departments most often performed this role. These external agents provided technical assistance, training, fidelity-monitoring, marketing, and other support for a defined period to implementing organizations (19).^1^ Such external support is one factor that was examined in the study described in this article.

Given the multiple program components and requirements for both instructors and organizational sponsors of CDSMP, the need for specific marketing or referral methods to attract participants into the program, and the specific funding needed to sustain it, this EBHP program can be considered a complex innovation. Embedding the program and sustaining it requires ongoing commitment by the organization to continue to invest in the training, materials, and outreach to keep the workshops filled and facilitated by instructors who meet the protocol requirements.

### Study purpose

The ARRA-funded national dissemination and implementation effort for CDSMP provides an opportunity to study the experiences of organizations and a dataset, which can be mined. This study uses that dataset as a starting point to identify a set of organizations that effectively implemented and sustained the CDSMP. To be considered successful, the organizations had to have offered at least four workshops in the 2-year timeframe, with a completion rate of 65% or higher. All organizations had to continue to offer the program at the time of the interviews (2013).

Key informant interviews with organizational managers responsible for the program provided qualitative data. Using a set of internal and external factors that previously have been identified in the literature as important facilitators, this study examined commonalities and differences among two different types of organizations implementing the CDSMP. It focused on common internal facilitators and also explored the type and perceived value of external support provided by a key state agent – the department charged with dissemination of the program.

The research question was:

- “What affected implementation success of the evidence-based CDSMP among eight organizations located in two states – examining a defined set of implementation factors (internal and external)?”

The purpose of this article is to offer insight on implementation of CDSMP from the organizational perspective. Understanding more about what factors or influences positively support organizations on the CDSMP implementation “journey” from adoption to sustainability can help identify what needs to be enhanced, what barriers exist, how some organizations have overcome these barriers, and what lessons they have learned. Such insight can help enhance external supports, such as policy, technical assistance, public health marketing, or fidelity-monitoring as well as clarify internal organizational elements that were important. This knowledge may help increase the likelihood that organizations will effectively implement and sustain the program.

## Materials and Methods

This mixed methods study examined implementation of CDSMP by eight organizations located in two states (identified as State #1 and State #2) and the support offered by their state agency to facilitate dissemination and implementation. The two states remain unnamed to protect the identity of the respondents. There were two types of organizations included in this sample: aging services organizations (ASOs), including three area agencies on aging and one other aging services provider, and health care organizations (HCOs), including three hospital/clinic systems and one health care center.

### Data sources and sample selection

The ARRA dataset (secondary data) and results from an electronic survey (primary data) were used to conduct several iterations of review in order to select the study sample of organizations. The ARRA dataset provided information, by state, on the number and type of organizations that participated in CDSMP implementation from 2010 to 2012 through the ARRA network. States were selected that had participated in a previous national EBHP initiative (from 2006 to 2007). This was done in order to maximize the likelihood that the state had invested time and resources to create structures or processes that fostered dissemination and implementation of these EBHP programs.

There were 24 states that participated in both the prior EBHP initiative and the ARRA grant. The pool was further narrowed to seven states that had at least six ASOs and HCOs. The ARRA dataset was then used to find organizations that met a set of criteria indicating implementation effectiveness for CDSMP. These criteria were: the organization offered at least four workshops within the 2 years of the ARRA initiative, had at least a 65% completion rate, and continued to offer the workshop in 2013. In addition, the CDSMP program manager within the organization had to have institutional memory of the implementation process. Institutional memory is defined as knowledge of the organization’s motivation, climate, and/or steps to beginning the program by virtue of being employed by the organization during the timeframe when this occurred.

### Key informants and interview protocols

The qualitative data source was comprised of 10 semi-structured interviews conducted by telephone by the investigator. The two types of key informants were: (1) state representatives who were the responsible managers for the CDSMP in their state, and (2) organizational representatives who were the managers of the CDSMP within their implementing organizations.

Two semi-structured interview protocols were designed with questions probing a set of pre-defined implementation factors, drawing from the work of Durlak and DuPre, Fixsen, Greenhalgh, and Damschroder (1–3, 20). The investigator pilot-tested the instrument with a CDSMP manager who did not participate in the study. The investigator also had the instrument reviewed by a national program manager providing technical support to CDSMP implementing organizations.

Many of the items on the interview protocol had adjectival responses scaled (best to worst) with a corresponding weight from +2 to −2. This five-point scale is consistent with the scale used by Damschroder (20).

Training in interview techniques was not required as the investigator was a seasoned interviewer, having conducted more than 100 interviews over 20 years of experience in health services evaluation – of both health services professionals as well as laypersons. Key informants provided verbal and written consent. The questions were provided to each key informant at least 1 week prior to the scheduled interview. Each interview took about an hour to conduct. Interviews were recorded and transcribed. The study was submitted to the University of North Carolina Institutional Review Board and determined to be exempt.

Factors probed in the state representative interviews were:

- Drivers of CDSMP dissemination and implementation in the state.
- Type and level of assistance provided to implementing organizations in the state.
- Peer support and communication – whether and how this was fostered within the state.

Factors probed in the organizational interviews included:

- Drivers for adoption within organization – the “will” to do this.
- Program fit.
- Ease of use of the protocol.
- Value.
- External support (particularly state agency support and peer networking).

State agency representatives were interviewed first (April/May 2013). This allowed the investigator to ask questions about the external context in which the implementing organizations had been operating. It provided a picture of the state’s activities in fostering CDSMP from the state’s perspective, prior to hearing from the organizations. The aging services division was the entity responsible for CDSMP dissemination within State #1. Two representatives from this agency participated in the key informant interview, including the program coordinator who had been in that role for 3 years and the director of the division. One representative from State #2 participated in the key informant interview. This individual had served as the program coordinator since 2007 and was from the department of public health.

Organizational representatives were interviewed second. There were eight organizational key informants (one per organization) who participated in interviews between June and August, 2013. All had been involved in the implementation of CDSMP for their organizations for at least 3 years and all were CDSMP Master Trainers.

## Results

The results from the state key informant interviews provide context and background to the organizational data and therefore are offered first.

### States’ perspectives – adoption and early efforts

The technical assistance and dissemination support to implementing organizations from State #1 focused on building the capacity and infrastructure for CDSMP. Drivers for the state included interest in helping elders to stay active and healthy-support for CDSMP and other EBHP programs were included in the State Plan. The state began offering “mini-grants” to aging services providers interested in CDSMP through a competitive application process. The state tapped into the existing network of Area Agencies on Aging. The primary support provided to implementing organizations were start-up grants (to cover workshop direct costs), Master Trainer and Peer Leader training sessions, and the CDSMP workbooks, which were to be given or loaned to participants. This state focused on having a corps of Master Trainers trained by Stanford University. The Master Trainers would then train workshop leaders.

Drivers for CDSMP came from the public health department in State #2. The attraction was the evidence base and the defined purpose/focus for this program, which emphasized personal engagement in one’s own health. This state contracted with external agencies to provide technical assistance and support to implementing CDSMP organizations. Through these agencies, the state provided training, workshop materials, marketing support, and fidelity-monitoring. The state also required implementing organizations to participate in peer collaboration and information sharing. Initially, this state paid for the license cost of each funded organization under ARRA. With state support the number of Master Trainers grew substantially.

Comments by these state representatives about early dissemination efforts included:

We had programs that encouraged health and wellness of seniors – but before 2006 people were not aware of CDSMP. We did not have this evidence-based program. There was only 1 Master Trainer in the whole state. We needed to build capacity and infrastructure. We started with natural partners who had an interest.

We included this kind of focus in our State Plan. There were major goals around empowering older people to stay active and healthy.

We had several organizations that were committed to evidence-based programing and knew about CDSMP. In fact our first training session was led by one of them. We created partnerships with these organizations and partnered with them very closely.

We found interest among organizations that had already had a successful track record of offering the program. It then grew very organically from one organization to another. As it [funding] was made available [for implementing organizations] we worked with organizations all over the state – rural, metro, etc.

One of these states contracted separately with a consultant agency to identify data elements for tracking and monitoring the program. Each funded CDSMP provider organization was required to submit data to the state office on these elements. In 2010, this state adopted a name for CDSMP to be used consistently statewide – this name was branded. In that same year, the state purchased a multi-organizational license for CDSMP for their state. This meant that many organizations that with their own single-organization licenses through Stanford switched to operate under the state’s license.

When asked about how information sharing was fostered among CDSMP implementing organizations, these state representatives described their role as conveners and facilitators – providing forums for these organizations to gather and communicate. This included regional meetings, newsletters, and electronic list-serves.

### States’ perspectives – implementation issues and sustainability

Since the ARRA funding ended, one state has focused on supporting the Master Trainers and ensuring fidelity-monitoring. This state also includes a calendar of workshop offerings on their government website as well as all the forms that CDSMP providers need. With the ARRA funding ended, the other state does not pay license costs for CDSMP providers, nor does it compensate organizations for training costs. The state representative explained that it has a philosophy of local authority and control and also that program sustainability requires embedding at the organizational level. Each organization is expected to create its own business plan to address CDSMP (as well as to support other health promotion, disability prevention programing). The state still provides some funding support to the external agency providing technical assistance and peer collaboration facilitation and marketing assistance. This state also maintains policy support for CDSMP (e.g., it is in the State Plan).

The state representatives offered the following insights about organizational implementation of CDSMP and sustainability:

I think that an across-the-board issue in implementation is staff turnover and agency redirection as a result … Any time [senior] management changes there is a question – will they see the value?

There is a very high investment upfront to become a CDSMP provider organization – heavy staff or volunteer training and certification, etc. That is also an ongoing issue – keeping the volunteers certified and active. They have many reasons why they might drop out including their own health issues.

One thing I’ve seen is if the organization doesn’t truly have the buy-in of the higher level administration, it will struggle when the funding ends. Grants are good for start-up, but a sustainability plan is needed.

### Organizational perspectives

The results from the electronic survey showed that most of the eight organizations (75%) had begun offering CDSMP between 2006 and 2009 (one began before 2006 and one started in 2010). All of the organizations had offered at least four CDSMP workshops in the 2-year time period. The range in number of workshops offered spanned from a low of 5 to a high of 21 in this time period. All eight organizations had an overall completion rate^2^ of 65% or higher, with a range from 66 to 78%. In addition, all of the organizations reported that they followed the program with fidelity. Thus the electronic survey confirmed these eight organizations met the criteria for inclusion – they represented a group of successful implementers with extensive experience.

#### Adoption and fit

The examination found agreement among the eight organizations on why the organization had adopted the CDSMP – citing alignment between the program and the organization’s purpose related to improving health and promoting better self-care. Many organizations had begun implementing the program prior to the ARRA grant funding in 2010 – thus the grant facilitated the work that this set of organizations had already begun (it was not the reason that they adopted the program). Informants frequently discussed the organizational leadership and internal champion for the program. This strong champion for the program (sometimes it was the respondent) was instrumental in getting their organization to adopt and implement CDSMP. For example, this comment was offered:

I was the champion for the program and then I influenced others. I think the evidence-based nature and availability of the training was what attracted me to CDSMP.

From the beginning and continuing through the time of the interviews – organizational respondents said that CDSMP was seen as a good fit. Even so, CDSMP was often discussed as being somewhat unusual compared to the organization’s other services. The structured protocol of CDSMP was what set this program apart from the organizations’ usual health education and wellness services.

This program is a great fit – it fits extremely well. However I would also say that (especially in the beginning) – in some ways it was new. It was outside the norm (the group workshop with a structured protocol) of what we typically did, how we typically provided education.

The program fits well with the organization. This is because the core concept of CDSMP is one of promoting the individual’s self-management.

Really this is a perfect fit with our organization. All of our volunteers are 55 and older and this program is designed around the idea of peer leaders. That fit perfectly.

**Figure 1:**
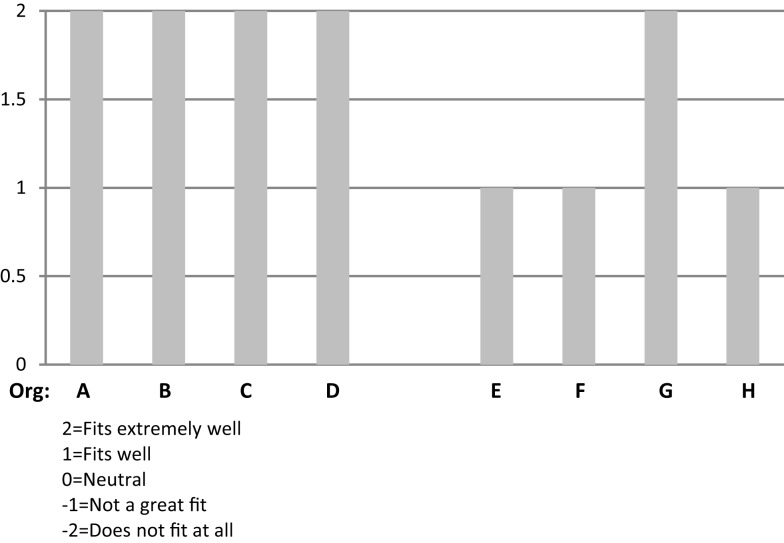
**Organizational respondents’ rating of CDSMP program “Fit” with their organization**.

Figure 1 provides a side-by-side comparison of the respondents’ ratings on “Fit” of CDSMP with their organizations.

Implementation of CDSMP requires a number of components (e.g., organizational licensure, instructor training and use of prescribed guidelines, session scripts and materials, and recruitment of participants). Despite this complexity, the program was often described by informants as relatively easy to adopt. This was true even though many of these organizations had not had any prior experience implementing an EBHP program.

**Figure 2:**
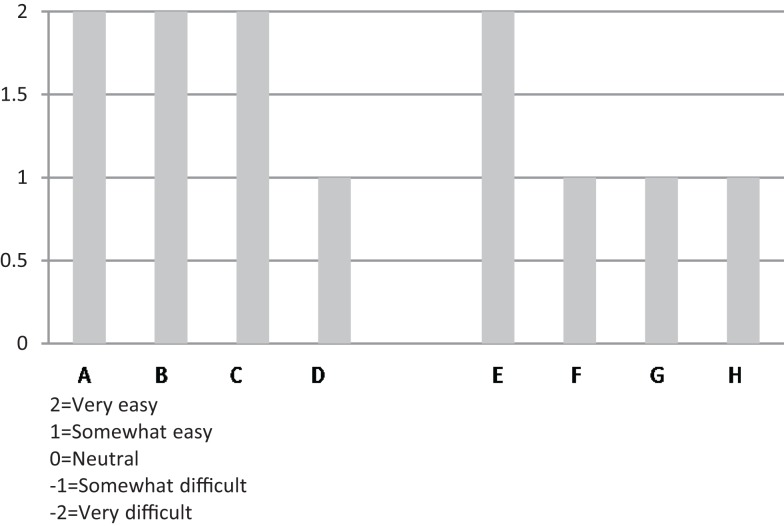
**Organizational respondents’ rating of “ease of use” of CDSMP protocol during their implementation experience**.

All informants mentioned that the heavily scripted workshop sessions, well-developed content of CDSMP, and required training assisted in implementation. This ease of use was noted by both experienced informants (e.g., community health educators who said they had used evidence-based protocols extensively) and by informants who said they had never used evidence-based protocols. Figure 2 provides a side-by-side comparison across the eight organizations on “ease of use” of the CDSMP protocol.

Even though we had never done an evidence-based class, I would say the protocol was very easy to follow. The guidelines were very clear.

I would say the protocol was very easy because of the partnership we had. When we first started we were under another license-holder’s license … they provided us with technical support and trainings, and the manuals. That made it easy.

This was somewhat easy in that it was scripted and heavily directed.

#### Value

**Figure 3:**
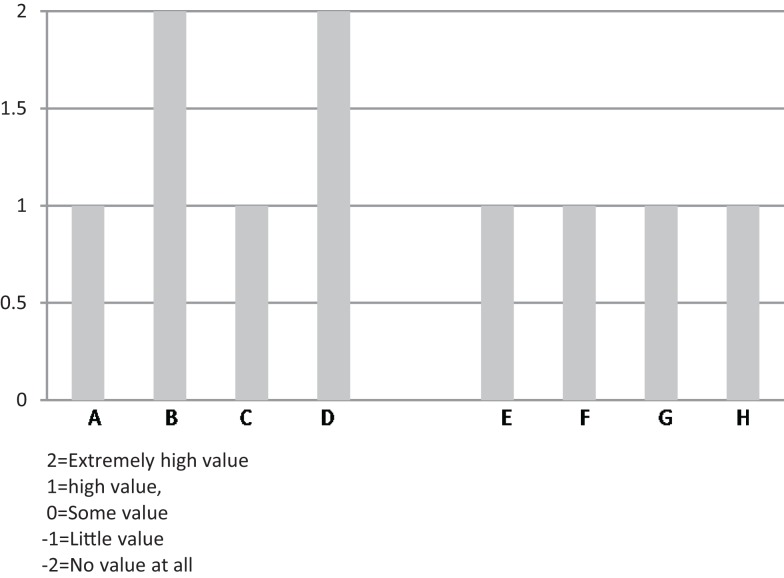
**Organizational respondents’ rating of “value of CDSMP” to their organizations**.

Comments about value focused on the participants and benefits they received from the program. Respondents talked about seeing participants make progress on their personal health goals and maintain a commitment to a healthier lifestyle. Respondents also discussed program value in terms of alignment with the future direction for the organization – many mentioned health care reform and the growing awareness of the need to achieve better population health management, prevent disability or decline (Figure 3). Comments are offered below:

This program is of extremely high value. It has proven results. It also attracts volunteers …

As we go down the health care reform path, I think this kind of program will be even more valued.

#### External support

One objective of this research was to assess the importance of external support to the organizations implementing CDSMP. The support from the state agency disseminating CDSMP was particularly probed, as the state department responsible for CDSMP dissemination was considered a key purveyor of the program.

Most of the informants indicated that their state agency had assisted with: funding, marketing, training, fidelity-monitoring, supplies, and peer networking. However, the way that these informants valued this support differed from “very helpful” to “neutral.” One reason given for the lukewarm rating given by a respondent was that the support from the state had diminished over time.

In addition to the support provided by the state, organizations named other sources of external support including: Stanford University (served as a source of information, provided supplies/materials, and guided fidelity-monitoring), local organizations such as libraries, senior housing facilities, senior centers and hospitals (helped with logistics, provided space, and helped with marketing), and the county health department (helped with peer leader training).

Respondents differed in how they experienced or perceived the level of peer support/collaboration their organizations received. Several remarked they did not receive much of this type of external support. A few said that there was extensive support and collaboration with similar organizations. This may indicate differences in the type or level of support offered – or it may be a function of the individual’s or organization’s commitment to and efforts around engaging in peer networks and collaborative activities. Comments included:

The peer collaboration is not growing. It was initially high, but as the program grew, it became minimal.

We have had modest peer collaboration.

There is extensive peer collaboration … We meet monthly via conference calls and share information and strategies … We’ve worked at making this CDSMP operate consistently across the state. We are doing fidelity monitoring the same way across the state and have set up a method to do that, as a peer group. We communicate regularly.

**Figure 4:**
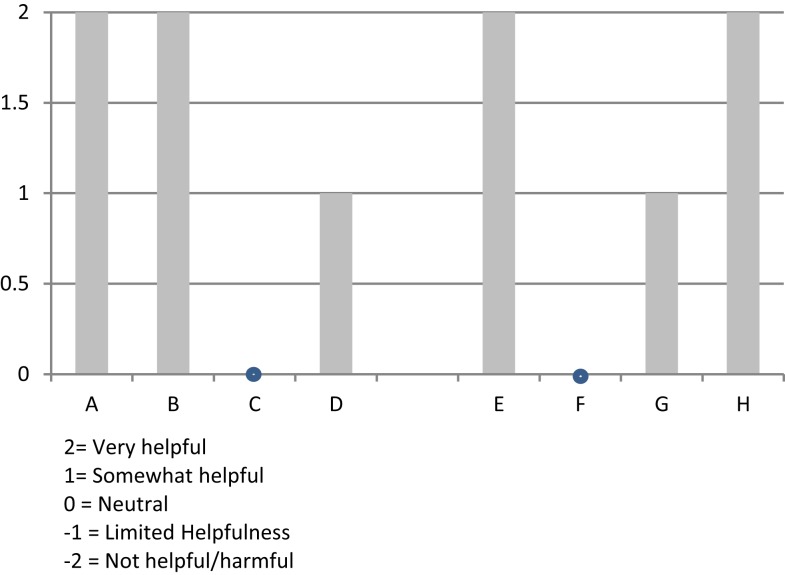
**Organizational respondents’ rating of “level of external support and helpfulness” during their implementation experience**.

A graphic depiction of the ratings from all eight organizations on the level and helpfulness of external support (a combined score from −2 to +2, corresponding to the adjectival responses) is shown in Figure 4.

### Challenges

Organizational key informants were asked about their implementation challenges. There was substantial consistency among this set of eight respondents about the challenges they faced in implementing CDSMP and in sustaining the program.

#### Recruitment/lack of demand

Recruiting participants and filling workshops was the number one challenge described by five of the eight organizational informants. Seven of the eight organizational informants said that it took “very significant effort” (−2) or “significant effort” (−1) to fill the workshops. Stanford fidelity guidelines recommend that class size be from 8 to 16 participants to optimize peer support and problem-solving. Six of the eight organizational representatives said that they have had to cancel a class at some point in time due to insufficient registration. These findings are consistent with other studies (19, 21).

The lack of demand for CDSMP was seen as being an effect of at least two things. First, very few individuals with chronic disease self-identify as needing the program – that is the individual hearing or reading about CDSMP does not interpret the program as being relevant for them. Marketing to consumers directly was challenging. This group of organizational managers instead often sought out other collaborative agencies, such as seniors center managers, senior housing facility managers, case managers, or health coaches to describe and promote the program as well as encourage participation among their clientele. Second, there was a lack of awareness on the part of physicians and other clinical providers about CDSMP and its’ benefit. The organizational informants said that they rarely had direct referrals from physicians to the CDSMP workshops (except where the program was a referral option within the health system’s medical information system).

Informants described extensive efforts to market the program and educate adults about the benefits of the program. The four aging service organizations more often described their “sales” and “outreach” efforts – going to senior centers, retirement housing facilities, doctor’s offices and putting up fliers, including information in newsletters, and networking with local social services agencies. The four HCOs more often described their internal health system connections as sources of referrals, including physicians working in the health system and health coaches.

Getting the workshops/classes filled is difficult – getting the number of participants we need to hold the class. They need it, but they don’t understand that – it has to be sold.

It takes significant effort – marketing and recruitment to fill the workshops.

We have a system of referrals within the clinic. If a provider wants to refer he or she can click on the classes we are offering through our electronic system – then we get the referral and follow-up.

**Figure 5:**
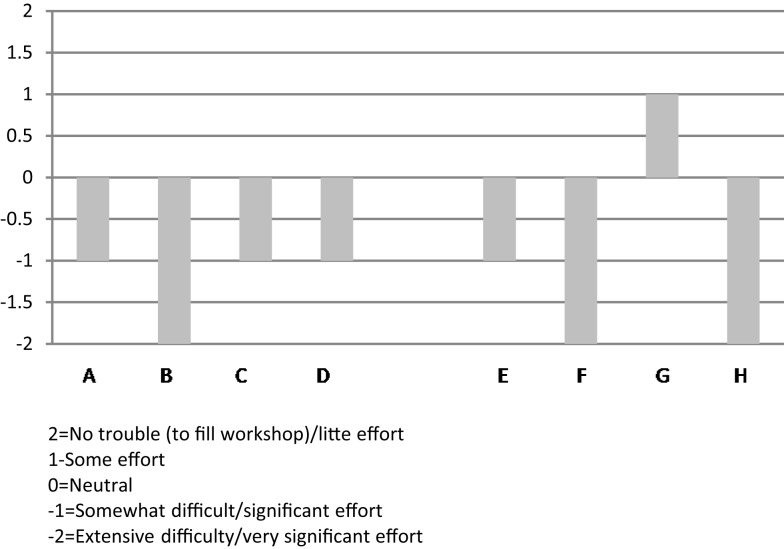
**Organizational respondents’ ratings on “demand/recruitment factor” – pertaining to the level of effort to fill CDSMP classes during their implementation of this program**.

A side-by-side comparison across the eight organizations of ratings on the “Demand/Recruitment” factor is shown in Figure 5.

#### Funding

Many of the key informants also discussed challenges with funding the CDSMP. The ASOs and HCOs differed in where they obtained funds to support the program. However, they were alike in commenting that funds received did not cover full costs.

Three of the aging services providers had partial funding of their CDSMP through Older American Act Title III-D funds. Donations and small fees as well as supplemental state grant funds were other sources of revenue to cover costs. One organization was covering the costs entirely out of their core operational budget – which also relies heavily on grants.

Three of the HCOs talked about the lack of external funding and hospital budget issues. These organizations discussed the shorter-term focus of their organizations where community health education is not seen as core – more of a community benefit. Therefore CDSMP and other health promotion, disease prevention programs are vulnerable to budget cuts. Despite this, one HCO respondent saw the potential for CDSMP. She said it was becoming more relevant for where the health care system is going in terms of accountability for population health. Despite their lack of current external funding, there was some optimism around the growing awareness and support for this type of program among the health care organizational respondents.

Without additional funding we can’t do this on an ongoing basis … I only expect to do 1 or 2 [workshops] this year. This is down from the 18 workshops we did in 2011 and 2012.

Hospitals are under a lot of budget restrictions. They are less able to provide this kind of community benefit now. We’ve had some reductions in staff in community health education. I don’t think this is self-sustaining – not so far.

I think there is some potential demand for this – under ACOs there is a commitment to population health and every member within their population. There is a basic level of service to be provided. CDSMP could be part of that.

### Organizational advice

Organizational informants reflected on lessons learned. They used various strategies to address implementation challenges or enhance their programs.

Strategies and advice included:

- Have a strong program champion internally.
- Build and maintain support at all levels internally especially senior administration and managers or clinical professionals who can serve as referral sources internally.
- Pursue a variety of ways to extend reach and improve visibility of CDSMP in order to build external referral sources and tap into collaborative resources (e.g., volunteers, building space for workshop locations, etc.).
- Recruit, train, and retain strong workshop leaders (staff or volunteers).
- Conduct ongoing marketing and outreach to make target population groups (potential participants) aware of the program.
- Measure results. Present a “return on investment” or value proposition to key stakeholders.

## Discussion

A study of eight organizations, purposively selected because of long-term successful experience with CDSMP, revealed that internal drivers and capability were more often discussed as facilitating successful implementation than external factors.

### Facilitating factors

Common facilitating factors for adoption and successful implementation of the CDSMP included:

- Program-to-organization fit.
- Organizational leadership.
- Training and well-developed materials.

These factors are consistent with other studies examining program implementation and sustainability. For example, a review of 19 empirical studies of health-related programs found the following factors to be important to the organizations (the study focused on organizations continuing the program at least 2 years following the ending of external funding): program champion, program fit with organizational mission, perceived value/benefits to clients, and support of stakeholders (22).

#### Program-to-organization fit

These eight organizational informants discussed alignment between the focus and purpose of CDSMP and the overall purpose or mission of their organizations as a facilitating factor in adoption and implementation effectiveness. Others studying implementation success have discussed the importance of fit between the innovation (program) and the organization – particularly the fit with the purpose or *values* of the organization (3, 4). This may be an important baseline criterion for an organization to consider when considering an EBHP program to adopt.

#### Organizational leadership

The managers responsible for CDSMP discussed both their own leadership as internal champions of the program, and the leadership from their supervisors, department directors, or senior executives – who demonstrated their support for CDSMP adoption and the implementation process. These administrators remained committed to offering the program even with limited funding.

Supportive leadership has been identified elsewhere as an organizational characteristic linked to successful implementation (23). The importance of champions and organizational leadership has been found to be a facilitator to health promotion practices being adopted, implemented, and maintained. For example, in a study of five Canadian provincial efforts to adopt a chronic disease prevention initiative, the research team found that there was “remarkable consistency in the top factors identified as facilitators and barriers to health promotion capacity building” [(7), p. 470]. Internal organizational factors were *most frequently mentioned* as facilitating implementation (more than external factors). Organizational respondents particularly noted the importance of having skilled, committed staff and supportive senior leadership (7). This may be another baseline criterion for organizations when considering EBHP program adoption.

#### Training and materials

Among these organizations, the CDSMP protocol and training materials were described as well-developed, easy-to-use, and excellent guides. These materials and training sessions worked well for both staff members and volunteers. Researchers of implementation effectiveness have discussed the importance of having quality tools and training (e.g., manuals, guides, worksheets, education, skills development, etc.) to support organizational performance and implementation effectiveness (23).

#### External support

External support also facilitated implementation among these eight organizations. Key external supports described as “very helpful” by these eight organizations were: (1) funding, (2) training and workshop materials, and (3) fidelity-monitoring. The value of peer support and collaboration varied among this set of respondents – for some organizations this type of support had been (and continued to be) very important. Other organizations had not participated and/or did not rely on peer support very much. These findings are consistent with other research identifying key supports for implementation, including the perceived benefits of using the EB program and collaborative technical assistance or program supports that are matched or tailored to the organization (12). This external support may be particularly important to the organization in the adoption and early implementation phases.

### Barriers and threats to sustainability

Common barriers and threats to sustainability included difficulties in recruiting participants, and lack of funding for the program – including lack of participant health insurance coverage for this type of EBHP program. It is likely that these challenges are linked.

#### Lack of demand

Recruiting participants to the workshops was a key challenge among the organizations in this study. Seven of the eight organizations said that getting participants into the workshops was the number one challenge. Organizations said that there is low awareness of the program among both the lay public and physicians – a key referral source.

The need for better marketing and distribution systems for public health programs has been identified elsewhere. In a study of 32 community-based prevention programs only modest penetration occurred in the marketplace, which limited impact. The researchers called for more effective approaches that “employ a reinforcing combination of both high-risk (targeting) and population-wide strategies” [(24), p. 571]. Others working in public health have pointed out the stark contrast between the sophistication of marketing and distribution systems for products and services in the business sector and the “unassigned, underemphasized, and underfunded” dissemination strategies in the public health sector [(25), p. 215].

#### Lack of funding/insurance

Since many people with chronic conditions have Medicare as their primary insurance, the fact that CDSMP is not covered by Medicare is an impediment.^3^ Medicare beneficiaries (and physicians) may believe that if a service or program is not covered by Medicare, then that service has not been shown to have enough benefit to the patient/consumer to warrant coverage. This has been shown to be true in other studies where the lack of insurance coverage contributed to underuse of proven services, such as secondary prevention programs in cardiac rehabilitation (26). Other researchers studying implementation and sustainability have noted that fiscal support is a critical external factor in some or all stages of adoption, implementation, and sustainability (6, 27).

While the focus or mission of health care and ASOs may be to assist individuals to improve or maintain health, they are reimbursed largely for addressing problems (after-the-fact), not preventing them. HCOs’ reimbursement comes primarily from illness care/treatment not prevention (28). For social service organizations such as area agencies on aging that provide direct services to elders, provision of services is primarily based on an older individual requesting help for an existing problem or need (relying on grant and OAA funding and skewed to those financially vulnerable). Thus, the CDSMP runs into the same challenge that many public health interventions face: lack of funding for prevention.

It is the author’s opinion, that without a regular source of funding or payment for service, the “value” of CDSMP has to be demonstrated one person and one provider at a time. The lack of payment for EBHP programs such as CDSMP may be interpreted by the lay public or by physicians as a signal that the program does not provide sufficient value in terms of health status improvement or effect. Given these forces at work, participation remains low and each referral/registration to a CDSMP workshop is hard-won. Organizations expend extra effort to get the program costs covered for those who do elect to participate. Without demand, there is little pressure to pay for these programs. Thus, the cycle perpetuates.

This issue goes beyond what the single manager within an implementing organization can address alone or even what a program coordinator at the state level can solve. It calls for a systems approach – where the stakeholders are aware of common overall objectives, their roles and boundaries in producing results, and the accountability of component parts to one another (29–31).

It is also important that policy and technical assistance is informed by and supportive of practice in the field. The Centers for Medicaid and Medicare Services (CMS) has commissioned studies of EBHP programs that include CDSMP, but notes challenges, particularly how to directly fund community-based wellness and prevention programs for this and other programs (32). CMS calls for more research to: “develop a sustainable framework for supporting a health ecosystem of community-based providers, while not exposing the Medicare program to undue risk” [(32), p. 72].

Meanwhile the infrastructure to support CDSMP may be eroding. The infrastructure investment under ARRA facilitated regional and local training, grew state, and organizational expertise on how to run these programs, fostered fidelity-monitoring peer collaboration and shared learning, produced Master Trainers and peer workshop leaders in every state, and engaged implementing organizations to commit to and market the program. As evidenced by the response from these eight organizations in just two states, these external supporting and infrastructure components seem to be shrinking.

### Limitations

The primary limitation of this study is due to there being a single investigator. A second researcher to review and confirm the categorization and coding of comments and to interpret the findings would add strength to this examination. This limitation was addressed into some degree by: (1) conversations with experts in the field who are familiar with CDSMP and its implementation history and challenges, (2) careful crafting of the key informant interview instruments, (3) feedback from the national dataset program manager familiar with organizations implementing this program and with state agents, and (4) review of published studies about CDSMP, particularly recent program evaluations to identify important external factors. Other limitations include the small sample size of organizations and the exclusive focus on “successful” implementing organizations. This research would be strengthened by examining additional organizations of many types, located within other states, and having variable success in implementation – using the same interview protocol including the scaled response options.

## Conclusion

Reflecting on the lessons learned from these eight successful CDSMP organizations, recommendations related to enhancing internal and external supports are offered.

Supportive elements for adoption and early implementation efforts that drove these organizations were very consistent, especially organizational leadership and the perceived value and fit of CDSMP with the mission and purpose of the organizations. Advice by this set of successful organizations included clarifying the benefit of the program using both participant and organizational metrics. Thus recommendations to enhance *internal* capability to support effective implementation include:

- Identify an internal program champion who has the ability to help drive adoption and ensure senior level buy-in and commitment to the program;
- Make the case that the program is a good fit with the organization’s mission and purpose;
- Clearly identify the value of the program in terms that make sense to key stakeholders – e.g., to the participant, organization, funders, and policymakers. Measure and report this value consistently and repeatedly to enhance demand and solidify the foundation of support – which will help raise awareness of the value and benefits of CDSMP in the local area and should help in referrals to the program.

External supports were also clearly important for adoption and early implementation efforts among these organizations. Training and fidelity-monitoring were especially noted, as was funding to get the program up and running. Organizations noted that they could have also used help building awareness of the program. Therefore, recommendations for program sustainability in terms of *external supports* include:

- Enhance supportive policy at the federal and state level for CDSMP and programs like it that focus on improving prevention and self-management behaviors of individuals with chronic conditions and engaging individuals in their own care through fostering organizational readiness;
- Maintain support for training and fidelity-monitoring as a funded external support that appears to be key to both implementation and sustainability;
- Improve visibility/public awareness of the program through national campaigns or other methods to lessen the burden on each organization to make this program known;
- Accelerate efforts to make benefit changes to the Medicare program to include CDSMP as a defined benefit for persons with chronic conditions to reduce the barriers around funding the program and to embed it in the fabric of the organizations that have done the hard work of adopting and implementing it.

Reaching individuals who can benefit from EBHP and disability prevention programs is an important public health goal. As health care and social support “systems” within the U.S. move haltingly forward toward more accountability for producing outcomes in health status among defined population (patient/client) groups, programs like CDSMP will become more relevant as a strategy for population health management. At that time, perhaps the program will be seen as a fundamental service of these organizations, with funding allocated through internal budgeting processes.

Until that time, organizations willing to adopt such programs must be supported effectively. Key external supports, such as training, materials, and funding provide the bedrock for dissemination and implementation [(11), p. 46]. National marketing campaigns or other external marketing supports are clearly needed. These findings are consistent with a more extensive review of activities at the state (intermediary agent) level on CDSMP diffusion and dissemination. In a final evaluation report about CDSMP to the Administration on Aging (focusing on states’ activities), the authors also recommended a “centralized or coordinated process for recruitment, intake, referral, and registration/enrollment” [(19), p. 97]. Both studies call for a coordinated, systems approach. Without this greater effort, individuals with chronic conditions, medical providers, and potential referral organizations within a given region are likely to remain unaware of the program and its value. This is a tremendous missed opportunity for public health and disability prevention.

Chronic disease represents one of the top public health issues domestically and globally. Stronger public policy to ensure there is an infrastructure to support EBHP programs that have demonstrated effectiveness with chronic disease populations should be a public health priority.

## Conflict of Interest Statement

The author declares that the research was conducted in the absence of any commercial or financial relationships that could be construed as a potential conflict of interest.

This paper is included in the Research Topic, “Evidence-Based Programming for Older Adults.” This Research Topic received partial funding from multiple government and private organizations/agencies; however, the views, findings, and conclusions in these articles are those of the authors and do not necessarily represent the official position of these organizations/agencies. All papers published in the Research Topic received peer review from members of the Frontiers in Public Health (Public Health Education and Promotion section) panel of Review Editors. Because this Research Topic represents work closely associated with a nationwide evidence-based movement in the US, many of the authors and/or Review Editors may have worked together previously in some fashion. Review Editors were purposively selected based on their expertise with evaluation and/or evidence-based programming for older adults. Review Editors were independent of named authors on any given article published in this volume.

## Footnotes

^1^It should be noted that organizations do not have to be a part of a federal initiative to implement CDSMP.

^2^Completion was defined as the participant completing 4 of the 6 sessions of CDSMP.

^3^The Medicare program does provide coverage for patient education and rehabilitation, most commonly for specific defined time periods and usually related to a new diagnosis, illness, surgical procedure, or injury, or an exacerbation of an existing condition/issue.
